# The role of muscular traction in the occurrence of skeletal relapse after advancement bilateral sagittal split osteotomy (BSSO): A systematic review

**DOI:** 10.1111/ocr.12488

**Published:** 2021-06-23

**Authors:** Maxim Van den Bempt, Shankeeth Vinayahalingam, Michael D. Han, Stefaan J. Bergé, Tong Xi

**Affiliations:** ^1^ Department of Oral and Maxillofacial Surgery Radboud University Nijmegen Medical Centre Nijmegen Netherlands; ^2^ Department of Oral and Maxillofacial Surgery University of Illinois at Chicago Chicago IL USA

**Keywords:** BSSO, muscle, orthognathic surgery, relapse, stability

## Abstract

The aim of this systematic review was (i) to determine the role of muscular traction in the occurrence of skeletal relapse after advancement BSSO and (ii) to investigate the effect of advancement BSSO on the perimandibular muscles. This systematic review reports in accordance with the recommendations proposed by the Preferred Reporting Items for Systematic Reviews and Meta‐Analyses (PRISMA) statement. Electronic database searches were performed in the databases MEDLINE, Embase and Cochrane Library. Inclusion criteria were as follows: assessment of relapse after advancement BSSO; assessment of morphological and functional change of the muscles after advancement BSSO; and clinical studies on human subjects. Exclusion criteria were as follows: surgery other than advancement BSSO; studies in which muscle activity/traction was not investigated; and case reports with a sample of five cases or fewer, review articles, meta‐analyses, letters, congress abstracts or commentaries. Of the initial 1006 unique articles, 11 studies were finally included. In four studies, an intervention involving the musculature was performed with subsequent assessment of skeletal relapse. The changes in the morphological and functional properties of the muscles after BSSO were studied in seven studies. The findings of this review demonstrate that the perimandibular musculature plays a role in skeletal relapse after advancement BSSO and may serve as a target for preventive strategies to reduce this complication. However, further research is necessary to (i) develop a better understanding of the role of each muscle group, (ii) to develop new therapeutic strategies and (iii) to define criteria that allow identification of patients at risk.

## 1 INTRODUCTION

The key factors that determine the success of orthognathic surgery include an accurate diagnosis, optimal treatment planning, accurate transfer of the treatment plan to the patient during surgery and the stability of the post‐operative results.^1^ With the introduction of three‐dimensional (3D) virtual surgical planning and the development of computer‐assisted techniques, the orthognathic treatment plan can be transferred to patients with high accuracy in all three dimensions.^2^, ^3^ However, long‐term stability remains a major concern after an advancement bilateral sagittal split osteotomy (BSSO).^4^, ^5^

Incidences of clinically significant skeletal relapse (SR), often defined as an SR larger than 2 mm, of up to 46% have been reported in the literature.^6^ The amount of long‐term SR after BSSO advancement varies between 1.5% and 50.3% of the initial advancement at B‐point in all patients.^4^, ^7^, ^8^ The aetiology of SR seems to be multifactorial, and several influencing factors, such as the amount of advancement,^4^, ^8^, ^9^, ^10^, ^11^, ^12^, ^13^, ^14^ type of fixation,^4^, ^7^, ^9^, ^13^, ^15^, ^16^ mandibular plane angle (MPA) ^4^, ^6^, ^11^, ^12^, ^14^ and control of the proximal segment ^4^, ^9^, ^13^, ^17^ have been identified.

The occurrence of SR has been largely attributed to increased soft tissue and muscular tension due to mandibular advancement.^18^ This may also explain the higher incidence of SR in patients with a high MPA, as the muscles of mastication are stretched in the ramus area when the proximal segment is rotated in the counter‐clockwise direction.^4^ Furthermore, this hypothesis can possibly explain the major differences in stability between rigid internal fixation (RIF) and wire fixation, as RIF might be more resistant to dorsal traction by the perimandibular muscles on the advanced mandibular segment. This muscle tension hypothesis was further investigated by Ellis and Carlson, who conducted an animal study on 10 rhesus monkeys in which the mandible was surgically advanced.^19^ In five of these animals, an additional myotomy of the suprahyoidal (SH) muscles was performed. In the non‐myotomy group, the mean SR was 13.19% or 0.66 mm (range: 0.01‐1.52 mm) after 6 weeks of maxillomandibular fixation, whereas the length of the mandible remained stable in each subject of the myotomy group. At 90 weeks after the release of maxillomandibular fixation, no further SR was observed in the control group, whereas a significant lengthening of the mandible by an average of 1.76 mm occurred in the myotomy group.

Considering the factors associated with the occurrence of SR, how the influence of all these factors may be explained by this muscle tension theory and the findings of the animal study by Ellis and Carlson, it can be suggested that muscular stretch may play a role in the occurrence of SR after BSSO advancement.

The primary aim of this systematic review was to determine the role of muscular traction in the occurrence of SR after the BSSO advancement. The secondary aim was to investigate the effect of BSSO advancement and the resulting dentoskeletal changes on the perimandibular muscles.

## 2 MATERIALS AND METHODS

This systematic review was conducted in accordance with the recommendations of the Preferred Reporting Items for Systematic Reviews and Meta‐Analyses (PRISMA) statement.^20^ The protocol for this systematic review was registered on the International Prospective Register of Systematic Reviews (PROSPERO).

### 2.1 Focused question

The review focused on the following research question: Does muscular activity/traction play a role in the occurrence of SR after advancement BSSO?

A secondary research question was as follows: Does BSSO advancement affect muscular activity/traction after surgery?

### 2.2 Search strategy

To identify the relevant studies, a systematic search was carried out in the MEDLINE, Embase and Cochrane Library databases using the modified PICOS strategy displayed in Table 1. All studies published until 1 March 2019 were considered. No language restrictions were imposed.

**TABLE 1:** PICOS search strategy and combinations for electronic database searching

Patient: BSSO Advancement	Keywords: Orthognathic OR Bimax* OR BSSO OR sagittal split osteotomy OR bilateral sagittal split osteotomy OR mandibular advancement OR jaw surgery
	MeSH terms: Osteotomy, Sagittal Split Ramus, OR Mandibular Advancement
	Emtree: 'bilateral sagittal split osteotomy'/exp OR ‘bilateral sagittal split ramus osteotomy’/exp OR ‘mandible osteotomy’/exp OR ‘mandibular advancement’/exp
Intervention/Indicator:Muscular traction/adaptation	Keywords: muscle* OR masseter OR pterigoid* OR temporal* OR suprahyoid* OR digastric* OR mylohoid* OR geniohyoid* OR geniogloss* OR strength OR stretch* OR tension OR traction OR influence
	MeSH terms: Masticatory Muscle OR Neck Muscles
	Emtree: 'masticatory muscle'/exp OR 'suprahyoid muscle'/exp OR 'digastric muscle'/exp OR 'mylohyoid muscle'/exp OR 'geniohyoid muscle'/exp OR 'genioglossus muscle'/exp OR 'muscle tone'/exp
Comparison Outcome	NS
	Keywords: stability OR stable OR relapse OR loss of correction OR survival
	MeSH terms: Recurrence
	Emtree: 'relapse'/exp OR 'recurrent disease'/exp OR 'recurrence risk'/exp OR 'survival'/exp
Study design	NS

Abbreviations: NS, not specified.

### 2.3 Study selection

Study selection was performed by two independent reviewers (VDB and SV). Disagreements regarding entry were resolved by consensus.

The inclusion criteria were as follows: (i) assessment of SR after BSSO advancement; (ii) assessment of morphological and functional changes in the perimandibular muscles after BSSO advancement; and (iii) clinical studies on human subjects.

Following the removal of duplicates, all articles were screened by title. Titles that were not relevant to this review were also excluded. Subsequently, the remaining articles were evaluated using an abstract. The following exclusion criteria were applied: (i) surgery not comprising BSSO advancement; (ii) studies in which muscle activity/traction was not investigated; and (iii) case reports with a sample of five cases or fewer, review articles, meta‐analyses, letters, congress abstracts or commentaries. The full texts of selected articles were included in this systematic review. In addition, the reference lists of the included studies were screened to identify additional publications eligible for inclusion.

### 2.4 Data extraction

For the analysis of the influence of the perimandibular muscles on SR, the primary outcome variable was SR, as measured by imaging. The primary predictor variable was perimandibular muscle action. To analyse the influence of BSSO advancement on the perimandibular muscles, the primary outcome measure was morphological changes of the perimandibular muscles. Secondary outcome measures included functional changes such as maximum and relative strength. The primary predictor variable was the degree of mandibular advancement.

Each study was evaluated for the following variables: year of publication, study design, sample size, age and sex of the participants, pre‐operative angle classification, treatment, fixation technique, type and duration of retention, assessed outcomes, method of outcome assessment, reported outcomes and findings. These data were extracted from each study by both reviewers (VDB and SV). In case of disagreement between the reviewers, a discussion was undertaken until an agreement was reached. Custom‐made forms were used for the data extraction.

### 2.5 Risk of bias assessment

The Risk Of Bias In Non‐randomized Studies of Interventions (ROBINS‐I) tool was used to assess the risk of bias in the included studies.^21^ This tool covers seven domains through which bias might be introduced, that is confounding, selection of participants, classification of interventions, deviations from intended interventions, missing data, measurement of outcomes and selection of the reported results. The domains were classified as low (‐), moderate (±), serious (+) or critical (++) risk of bias.

The two reviewers (VDB and SV) independently assessed the risk of bias in the included studies. Disagreements regarding entry were resolved by discussion.

When the risk of bias could not be assessed properly due to lack of information in the published paper, an attempt was made to contact the corresponding author for clarification.

### 2.6 Data synthesis

The high heterogeneity of the reported outcome measures did not allow the quantitative synthesis of the data retrieved from the included studies. Therefore, a qualitative synthesis of the results was performed.

## 3 RESULTS

### 3.1 Search results

**FIGURE 1:**
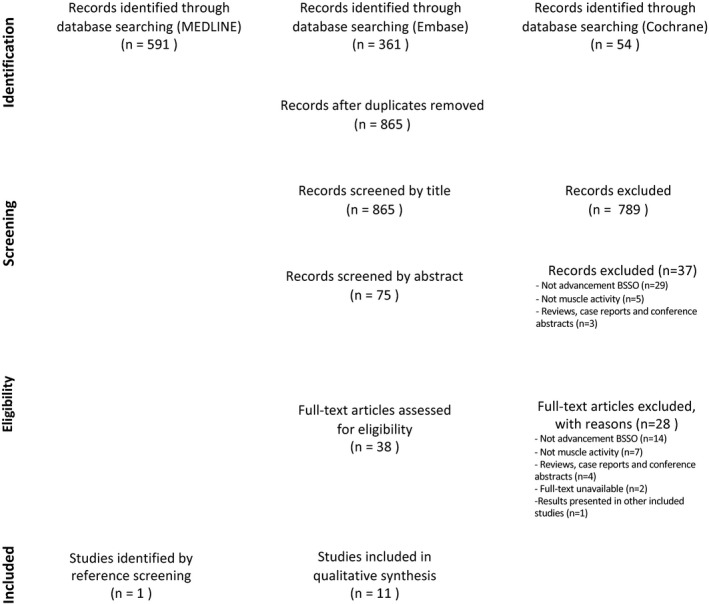
PRISMA flowchart

The process of selection and inclusion of articles is illustrated in Figure 1. The initial searches of the MEDLINE, Embase and Cochrane databases yielded 591, 361 and 54 studies, respectively. All 1006 records were checked for duplicates. After removal of duplicates, the remaining articles (n = 865) were screened by title, uncovering 75 potentially eligible articles. Of these 75 articles, 37 articles were excluded after reading the abstract against the preset exclusion criteria: surgery other than BSSO advancement (n = 29); studies in which muscle activity/traction was not investigated (n = 5); and case reports with a sample of five cases or fewer, review articles, meta‐analyses, letters, congress abstracts or commentaries (n = 3).

For the remaining 38 articles, the full text was retrieved and analysed in full detail. Twenty‐eight articles were excluded based on the full text for the following reasons: surgery other than BSSO advancement (n = 14); studies in which muscle activity/traction was not investigated (n = 7); case reports with a sample of five cases or fewer, article reviews, meta‐analyses, letters, congress abstracts or commentaries (n = 4), full text unavailable (n = 2), and results presented in other included articles (n = 1). The remaining 10 articles were included in the qualitative synthesis.^22^, ^23^, ^24^, ^25^, ^26^, ^27^, ^28^, ^29^, ^30^, ^31^

Screening of the reference lists of included studies yielded one additional study that met the inclusion criteria.^32^ A total of 11 articles fulfilled the inclusion criteria and were included in the final qualitative synthesis.^22^, ^23^, ^24^, ^25^, ^26^, ^27^, ^28^, ^29^, ^30^, ^31^, ^32^

### 3.2 Study characteristics

The characteristics of each included study are listed in Table 2.

**TABLE 2:** Characteristics of included studies

Author, Publication Year	Study design	Sample Size	Male/Female	Mean age (range)	Diagnosis	Treatment	Fixation technique	Retention	Outcome	Outcome assessment
Mücke T. 2016^22^	CCT			31	Class II div. 2		Miniplates	NS	Relapse	Lateral cephalograms
Group I		8	1/7			BSSO +BTA				
Group II		24	8/16			BSSO				
Van der Linden C. 2015^23^	Retrospective study				Class II		Bicortical screws	4 wk (Elastics)	Relapse	Lateral cephalograms
Group I		29	10/19	23(14‐46)		BSSO +genioplasty				
Group II		29	9/20	24(15‐46)		BSSO				
Beukes J. 2013^31^	Retrospective study				Class II		Bicortical screws	4 wk (Elastics)	Relapse	Lateral cephalograms
Group I		25	9/16	23 (15‐43)		BSSO +stripping				
Group II		25	7/18	27 (15‐47)		BSSO				
Dicker G. 2012^29^	CCT	16	8/8	27 (16‐45)	Class II		Bicortical screws	None	Muscle adaptation	MRI, lateral cephalograms
Group I		8			MPA <39°	BSSO				
Group II		8			MPA > = 39°	Bimax				
Dicker G. 2012^32^	CCT	16	8/8	27 (16‐45)	Class II		Bicortical screws	None	Muscle adaptation	MRI, lateral cephalograms
Group I		8			MPA < 39°	BSSO				
Group II		8			MPA > = 39°	Bimax				
Di Palma E. 2009^26^	CCT	19	9/10	(17‐34)			NS	NS	Muscle adaptation	EMG
Group I		4			Class II	BSSO				
Group II		15			Class III	Bimax				
Dicker G. 2007^30^	Case series	12	5/7	31 (18‐45)	Class II		Bicortical screws	None	Muscle adaptation	MRI
Group I		5			Short face	BSSO				
Group II		7			Long face	Bimax				
Eggensperger N. 2005^27^	Case series	15	4/11	21 (17‐31)	Class II	BSSO	Bicortical screws	4‐6 d (Rigid)	Relapse	Lateral cephalograms

Eckardt L. 1997^24^	CCT	52	NS	22 (3.1)			Bicortical screws	7‐10 d (Rigid)	Muscle adaptation	EMG
Group I		20			Class I	None				
Group II		15			Class II	BSSO				
Group III		17			Class III	Bimax				
Wessberg G. 1982^28^	retrospective study	16	NS	NS	Class II		Wires	6 wk (Rigid)	Relapse	Lateral cephalograms
Group I		8				BSSO +myotomy				
Group II		8				BSSO				
Wessberg G. 1981^25^	CCT	15	NS	adult			Wires	NS		EMG, Lateral cephalograms, Kinesiography
Group I		10			Class I	None				
Group II		5			Class II	BSSO				

Abbreviations: BTA, botulinum toxin type A; CCT, clinical controlled trail; EMG, electromyography; MRI, magnetic resonance imaging; NS, not specified.

Eight of the 11 included studies had a prospective study design,^22^, ^24^, ^25^, ^26^, ^27^, ^29^, ^30^, ^32^ of which six were controlled clinical trials ^22^, ^24^, ^25^, ^26^, ^29^, ^32^ and two were case series.^27^, ^30^ The three remaining articles were retrospective observational studies.^23^, ^28^, ^31^

A total of 273 participants were included in this systematic review. The number of subjects in each study ranged from 12 to 58.^23^, ^30^ In three articles from one research group, the same subjects were enrolled in multiple studies^29^, ^30^, ^32^; however, none of these studies were excluded because each investigated different outcome measures. The overall mean age ranged from 21 to 31 years.^22^, ^27^, ^30^ The mean age was not mentioned or calculable in three of the 11 included studies.^25^, ^26^, ^28^

A total of 211 subjects from all 11 studies were diagnosed with Angle class II malocclusion. In one study on 32 subjects, class II malocclusion was further specified to a class II division 2.^22^ In two of the 11 studies, class III subjects (n = 32) were included.^24^, ^26^ Two studies had an untreated control group consisting of class I subjects (n = 30).^24^, ^25^

A total of 203 subjects were treated with BSSO, whereas 40 subjects underwent bimaxillary surgery. Of the 203 subjects treated with BSSO, 70 in four studies had an additional intervention affecting the perimandibular musculature.^22^, ^23^, ^28^, ^31^

In eight studies,^22^, ^23^, ^24^, ^27^, ^29^, ^30^, ^31^, ^32^ rigid internal fixation was used, whereas in two studies ^25^, ^28^ osteosynthesis was performed using wires. The fixation technique was not specified in one study.^26^ In three studies,^24^, ^27^, ^28^ rigid intermaxillary fixation was used for post‐operative retention, whereas in two studies ^23^, ^31^ guiding elastics were applied. In three other studies, no post‐operative retention was used.^29^, ^30^, ^32^ The post‐operative retention protocol was not specified in three studies.^22^, ^25^, ^26^

Four of the 11 included studies aimed to investigate the effect of muscular activity on the occurrence of SR.^22^, ^23^, ^28^, ^31^ The seven other studies focused on the adaptation of muscles after BSSO.^24^, ^25^, ^26^, ^27^, ^29^, ^30^, ^32^ Of these seven studies, four studies investigated the morphological changes of the muscles,^27^, ^29^, ^30^, ^32^ whereas the three remaining studies investigated the functional changes.^24^, ^25^, ^26^

### 3.3 Risk of bias assessment

The quality of the included studies according to the ROBINS‐I tool for assessing the risk of bias in non‐randomized studies of interventions is shown in Table 3. There was an overall low risk of bias due to the classification of interventions or deviations in the intended interventions. However, as the treatment (BSSO or bimaxillary surgery) was not specified for each group in all studies, and the outcome assessment was not blinded, the risk of bias due to confounding factors and the measurement of the outcomes was considered moderate. The risk of bias due to missing data was also moderate as a result of dropouts during follow‐up in some studies. Due to the fact that the amount of advancement was often not reported, there could be a significant risk of bias due to the selection of the reported outcome. Finally, the most serious risk of bias was found in the selection of the participants due to either participant selection after the intervention or lack of a detailed description of the protocol for participant selection.

**TABLE 3:** Risk of bias

Risk of bias in/due to	Mücke T. 2016^22^	Van der Linden C. 2015^23^	Beukes J. 2013^31^	Dicker G. 2012^29^	Dicker G. 2012^32^	Di Palma E. 2009^26^	Dicker G. 2007^30^	Eggensperger N. 2005^27^	Eckardt L. 1997^24^	Wessberg G. 1982^28^	Wessberg G. 1981^25^	Risk of Bias
Confounding	+	‐	‐	‐/+	‐/+	+/‐	‐/+	‐	+/‐	+/‐	‐	+/‐
selection of participants	‐	++	‐	‐	‐	+/‐	‐	‐	+	++	+	+
classification of interventions	‐	‐	‐	‐	‐	‐	‐	‐	‐	‐	‐	‐
deviations of the intended interventions	‐	‐	‐	‐	‐	‐	‐	‐	‐	‐	‐	‐
missing data	‐	‐	‐	+/‐	+/‐	‐	+/‐	++	‐	‐	‐	+/‐
measurement of outcomes	+/‐	+/‐	+/‐	+/‐	+/‐	+/‐	+/‐	‐	+/‐	+/‐	+/‐	+/‐
selection of the reported result	‐	‐	‐	+/‐	+/‐	+/‐	+/‐	‐	+	+	‐	+
Overall risk of bias	+/‐	+/‐	+/‐	+/‐	+/‐	+/‐	+/‐	+/‐	+/‐	+	+/‐	+/‐

Abbreviations: ‐, low; +, serious; +/‐, moderate; ++, critical.

### 3.4 Perimandibular muscles and relapse

Four studies assessed the effect of interventions on the occurrence of SR.^22^, ^23^, ^28^, ^31^ The characteristics of these studies are summarized in Table 4.

**TABLE 4:** Perimandibular muscles and relapse

Author, Publication Year	Follow‐up	Group I (study group)				Group II (control group)				Findings
		N	Treatment	Adv.	Relapse	N	Treatment	Adv.	Relapse	
Mücke T. 2016^22^	1 y	8	BSSO +BTA mylohyoid muscle	2.8 mm (B)	−0.1 mm (B)	24	BSSO	1.3 mm (B)	−0.4 mm (B)	Significantly less horizontal relapse in group I compared to group II
				2.9 mm (Pg)	0.0 mm (Pg)			1.5 mm (Pg)	0.0 mm (Pg)	
				4.2° (SNB)	0.0 (SNB)			2.1 (SNB)	−0.6 (SNB)	
				1.1° (SNPg)	0.0 (SNPg)			1.5 (SNPg)	−1.0 (SNPg)	
Van der Linden C. 2015^23^	10 m	29	BSSO +Adv. genioplasty	5.6 mm (B)	−0.2 mm (B)	29	BSSO	5.2 mm (B)	−0.8 mm (B)	No significant difference in relapse between groups
				10.7 mm (Pg)	−0.9 mm (Pg)			4.9 mm (Pg)	−0.5 mm (Pg)	
				10.8 mm (Me)	−0.7 mm (Me)			4.8 mm (Me)	−0.5 mm (Me)	
Beukes J. 2013^31^	8 m	25	BSSO +stripping MPM & SML	4.71 mm (B)	+0.20 mm (B)	25	BSSO	5.36 mm (B)	−1.18 mm (B)	Significantly less horizontal relapse in group I compared to group II
				4.61 mm (Pg)	+0.05 mm (Pg)			5.12 mm (Pg)	−0.92 mm (Pg)	
				4.62 mm (Me)	+0.54 mm (Me)			5.05 mm (Me)	−0.89 mm (Me)	
Wessberg G. 1982^28^	24 m	8	BSSO +suprahyoid myotomy (geniohyoid and anterior digastric muscles)	21.3% (H‐Me)	48.5% (B)	8	BSSO	18.5% (H‐Me)	43.1% (B)	No significant correlation between post‐operative stretch of the SH musculature (T0‐T1) and total horizontal SR (T1‐T2)

Abbreviations: B, B‐point; BTA, botulinum toxin type A; H, hyoid; Me, menton; MPM, medial pterygoid muscle; Pg, Pogonion; SML, stylomandibular ligament; SNB, sella–nasion–B‐point angle; SNPg, sella–nasion–pogonion angle.

A total of 156 subjects with class II malocclusion were treated with BSSO advancement.^22^, ^23^, ^28^, ^31^ In 70 subjects, one of the following additional interventions was performed: botulinum toxin type A (BTA) injection in the mylohyoid muscle in eight subjects,^22^ advancement genioplasty in 29 subjects,^23^ stripping of the medial pterygoid muscle (MPM) and the stylomandibular ligament in 25 subjects,^31^ and myotomy of the geniohyoid and anterior digastric muscles in eight subjects.^28^

In all studies, the intervention group was compared to a control group of subjects who underwent solitary BSSO advancement. The amount of advancement and SR was assessed using lateral cephalograms. The mean follow‐up period ranged from 8 to 24 months post‐operatively.^22^, ^28^

The first study was published in 1982 by Wessberg et al^28^ The subjects in the treatment group (n = 8) underwent a BSSO advancement with additional myotomy of the geniohyoid and anterior digastric muscles, whereas those in the control group (n = 8) underwent a BSSO advancement without myotomy. After surgery, the hyoid‐to‐menton distance (H‐Me) increased by 21.3% in the treatment group and 18.5% in the control group. The horizontal SR at the B‐point was 48.5% and 43.1%, respectively, after a mean follow‐up of 24 months. Statistical analysis revealed no difference in SH stretch or SR between the groups. Furthermore, no correlation between SH stretch and SR was found in any group. The risk of bias assessment revealed a critical risk of bias in the selection of the participants in this study, as the 16 subjects were selected from a previous study population consisting of 87 cases.^33^

In 2015, Van der Linden et al compared a group of 29 subjects who underwent BSSO with additional advancement genioplasty to a control group (n = 29) treated with solitary BSSO.^23^ The advancement measured at the B‐point was 5.6 mm in the genioplasty group and 5.2 mm in the control group. However, the advancement measured at menton was 10.8 mm and 4.8 mm, respectively. Statistical analysis showed no significant difference in SR between the two groups after 10 months of follow‐up.

A third study, published by Mücke et al^22^ in 2016, introduced a new approach to alter muscular traction after BSSO. Thirty‐two subjects diagnosed with a class II division II malocclusion and a deep bite were included in this prospective study. The treatment group consisted of eight subjects with a high muscle tone of the mylohyoid muscle at pre‐operative assessment and were treated with BSSO and injection of BTA in the mylohyoid muscle. The subjects in the control group (n = 24) were treated with BSSO alone. Statistical analyses demonstrated significantly less SR in the treatment group at the one‐year follow‐up.

In addition to studies focusing on the SH musculature, Buekes et al^31^ investigated the role of the MPM and the stylomandibular ligament in the occurrence of SR. In the treatment group (n = 25), the MPM and the stylomandibular ligament were stripped of the medial surface of both the distal and proximal mandibular segments, whereas in the control group (n = 25), these structures were left attached. At 6‐16 months of follow‐up, significantly less SR occurred in the treatment group.

### 3.5 Morphological and functional changes of the perimandibular muscles

Morphological and functional changes in the perimandibular muscles after orthognathic surgery were investigated in four and three studies, respectively.^24^, ^25^, ^26^, ^27^, ^29^, ^30^, ^32^ The characteristics and main findings of these studies are summarized in Table 5.

**TABLE 5:** Morphological and functional changes of the perimandibular muscles

Author, Publication Year	Follow‐up	Outcome assessed	Group I							Group II (if applicable)					Findings
			N	Condition; Treatment	Results					N	Condition; Treatment	Results			
Dicker G. 2012^29^	28 m	Static joint reaction force: MM, MPM	8	MPA <39°; BSSO	SJRF pre/post: 214N/232N					8	MPA >39°; Bimax	SJRF pre/post: 239N/250N			Only small changes in static joint reaction force were observed in both groups
Dicker G. 2012^32^	28 m	Direction: MM, MPM	8	MPA <39°; BSSO	No significant directional changes					8	MPA >39°; Bimax	Significant change in sagittal direction of MM and MPM			Directional changes occurred in group II.
Di Palma E. 2009 ^26^	6‐8 m	EMG: MM, ATM	19	Class II/III BSSO/Bimax	Pre‐operative			Post‐operative	/		/	/			Non‐significant improvement of POCm, POCt and TC after surgery
					POCm: 81.94%			POCm: 84.65%							
					POCt: 84.18%			POCt: 86.43%							
					TC: 89.29%			TC: 89.29%							
Dicker G. 2007^30^	18 m	CSA and volume: MM, MPM	5	Class II SF; BSSO	CSA MM/MPM: −10,96%/+0.73%				7		Class II LF; Bimax	CSA MM/MPM: −18.09%/−13.59%			Significant decline in jaw muscle CSA and volume after BSSO advancement
					Volume MM/MPM: −12.17%/−7.55%										
												Volume MM/MPM: −18.67%/−18.33%			
Eggensperger N. 2005 ^27^	12 y	SH length and relapse	15	BSSO	Advancement		Relapse:		/		/	/			Significant correlation between suprahyoid stretch (T1‐T4) and total horizontal relapse (T1‐T4)
					4.1 mm (B)		−2.0 mm (B)								
					4.9 mm (Pg)		−2.5 mm (Pg)								
					4.3 mm (Me)		−1.8 mm (Me)								
					1.0 mm (H)		−3.3 mm (H)								
Eckardt L. 1997 ^24^	NA	EMG: MM	15	Class II; BSSO	Preop: Divergent EMG in 6 points				17		Class III; Bimax	Preop: Divergent EMG in 4 points			Class II: Normalisation of EMG Class III: Slight or no change in EMG
					Postop:Divergent EMG in 1 point (compared to eugnathic controls)										
												Postop: Divergent EMG in 3 points (compared to eugnathic controls)			
Wessberg G. 1981 ^25^	3 m	EMG and Kinesiography: MM, ATM, PTM, MPM	5	Class II; BSSO	CRPM	PRPM			10		Class I; None	CRPM	PRPM	No significant change in EMG and IOS after BSSO	
					IOS: No change	IOS: No change						IOS: +0.3	IOS: −0.2		
					EMG: −0.1	EMG: No change									
												EMG: No change	EMG: +0.1		

Abbreviations: ATM, anterior temporal muscle; Bimax, bimaxillary surgery; CRPM, clinical rest position of the mandible; CSA, cross‐sectional area; EMG, electromyography; H, hyoid; IOS, interocclusal space; LF, long face; Me, menton; MM, masseter muscle; MPA, mandibular plane angle; MPM, medial pterygoid muscle; N, Newton; percentage overlapping coefficient (masseter muscle/anterior temporal muscle); Pg, Pogonion; POC(m/t); PRPM, postural rest position of the mandible; SF, short face; SJRF, static joint reaction force; TC, torque coefficient.

In 2015, Eggensperger et al^27^ published a long‐term follow‐up study focusing on the post‐operative stretch and adaptation of the SH muscles in relation to the pharyngeal airway space and SR. Statistical analyses showed only a weak correlation between initial SH stretch and long‐term SR. However, there was a significant correlation between muscle complex lengthening during post‐operative follow‐up and SR. The morphological changes of the masseter muscle and the MPM after BSSO were investigated by Dicker et al.^29^, ^30^, ^32^ In the first study, MRI was used to assess the volume and maximal cross‐sectional area of these muscles pre‐ and post‐operatively in 12 retrognathic subjects undergoing BSSO advancement.^30^ The results showed a significant decrease, up to 18%, in both volume and maximum cross‐sectional area of the jaw muscles 10‐48 months post‐operatively. A second study was performed by the same research group to evaluate the change in muscle direction and moment arms of bite force^32^ and showed a significant change in the vertical direction of both the MMs and MPMs in the sagittal plane among the subgroup of subjects with an MPA greater than 39° treated with bimaxillary surgery. In a third study, data from the same subjects were used to generate a biomechanical model to assess the muscle forces on the condyle and the corresponding joint reaction force.^29^ The findings of this study showed only minor increases in the joint reaction force after surgery.

Wessberg and Epker evaluated the influence of the modified sagittal split technique, as described by Epker,^34^ on masticatory muscle function using EMG and kinesiometry.^25^ The results showed no significant changes in the interocclusal space or EMG activity of the masticatory muscles three months after surgery compared to the pre‐operative measurements. In a second study, the excitation pattern of the MM in both groups of treated subjects, Angle class II (treated with BSSO) and class III (treated with bimaxillary surgery), was compared to eugnathic controls (untreated) before and after surgery.^24^ The initial pattern differed from the eugnathic controls in 6/16 points in class II and 4/16 points in class III groups, respectively. After treatment, the 1/16 and 3/16 points differed from the controls. In the third study,^26^ four class II subjects treated with BSSO and 15 class III subjects treated with bimaxillary surgery were included. EMG activity was registered in the anterior temporal muscle and MM pre‐operatively and at 6‐8 months post‐operatively. The results showed a non‐significant improvement in the percentage overlapping coefficient (POC), a measure of the symmetry of EMG activity of MMs (*P* = .072) and anterior temporal muscles (*P* = .125). Furthermore, a non‐significant improvement in the torque coefficient (TC), a measure of lateral deviation of the mandible by unbalanced EMG activity in the MM and contralateral anterior temporal muscle couples, was found.

## 4 DISCUSSION

### 4.1 Perimandibular muscles and relapse after BSSO advancement

The first study investigating the role of muscular traction in the occurrence of SR after advancement BSSO was published in 1982 by Wessberg et al.^28^ The authors concluded that SR is not significantly altered by SH myotomy; therefore, the SH muscles cannot be primarily responsible for SR after BSSO advancement. This conclusion is supported by the finding of Van der Linden et al that additional stretch of the SH muscles, by simultaneous advancement genioplasty, was not associated with an increase in the occurrence of SR.^23^

Although the results of these two studies ^23^, ^28^ seem to disprove the theorem that the stretching of the suprahyoid musculature, resulting in a posteriorly oriented force on the advanced distal segment of the mandible, contributes to the occurrence of SR, Mücke et al introduced BTA as a new approach to alter muscular traction after BSSO with promising results with regard to SR, as none of the subjects in the treatment group (n = 8) showed SR at follow‐up, whereas in the control group, SR occurred in more than 50% of patients.^22^

In an effort to explain the discrepancies in the findings of these three studies, differences in methodology, as well as the biological and anatomical consequences of the different interventions, should be considered. With regard to patient selection, it should be noted that the treatment group in the study by Mücke et al^22^ consisted of patients with pre‐operative palpable muscle tension and a dentofacial profile at risk for SR, whereas Wessberg et al^28^ and Van der^23^ Linden et al included a less specific population. Wessberg et al did not report the initial advancement, which should be considered an important limitation because the amount of advancement is associated with the occurrence of SR.^4^ However, the most important limitation of this study is that wire osteosynthesis was used in these patients, which limits the applicability of these results to BSSO with rigid internal fixation.^23^

A second possible explanation for the inconclusive results can be found in the anatomy of the SH musculature. Therefore, it should be noted that the interventions studied by Wessberg et al^25^ and Van der Linden et al^23^ are limited to the muscle fibres inserted at the chin, whereas the more posterior fibres of the mylohyoid muscle remain unaffected. In contrast, Mücke et al^22^ injected botulinum toxin A into the entire mylohyoid muscle, also relieving the tension of the posterior muscle fibres. As these shorter, posterior fibres are prone to a proportionally higher amount of stretch compared to the longer anterior fibres, their share in the total retraction force on the advanced segment should not be underestimated. However, this hypothesis has not been reported or tested in the currently available literature and should be the subject of future research.

A third factor that should be considered when interpreting the results of these studies is the biology of muscle regeneration. Although it seems plausible that posterior traction exerted by muscle fibres inserted at the chin can be permanently relieved by performing a myotomy, the regenerative potential of skeletal muscle should not be neglected. Three phases have been identified in the healing of a skeletal muscle injury: (i) destruction phase, (ii) repair phase and (iii) remodelling phase.^35^, ^36^ In the remodelling phase, contraction of scar tissue occurs, pulling both ends of the intact muscle fibres back together, which might have contributed to the posterior traction exerted by the SH muscles.

In addition to studies focusing on the SH musculature, Buekes et al^31^ investigated the role of the MPM and the stylomandibular ligament in the occurrence of SR. Significantly less SR occurred if the MPM and the stylomandibular ligament were stripped of the proximal mandibular segment during BSSO advancement.

The evidence provided by the studies in this review indicates that the perimandibular musculature might serve as a possible target to prevent or reduce SR after the advancement of BSSO. However, it has to be emphasized that the available evidence is limited, and further research will be necessary to validate the benefit of current interventions in different subgroups of subjects in well‐designed clinical trials. Furthermore, a better understanding of the role of each muscle in the occurrence of SR after surgical correction of distinct dentofacial subtypes will enable the development of new techniques to limit post‐operative SR in specific subject groups.

### 4.2 Morphological changes of the perimandibular muscles

The effect of BSSO advancement on the morphology of the perimandibular musculature was first investigated by Eggensperger et al^27^ in 2005. Although only a weak correlation was found between the initial SH stretch and SR, there was a significant correlation between post‐operative lengthening and SR. These findings can be explained by the observations of Reynolds et al in an animal study on rhesus monkeys, showing that lengthening of the SH muscles occurs at the muscle‐bone and muscle‐tendon interfaces if the mandible is advanced not more than 5 mm; lengthening of the muscle belly was noted after advancements of more than 5 mm.^37^ Based on the findings of both studies, it appears that the continuous posterior movement of the hyoid bone lengthens the SH musculature by stretching the muscle‐bone and muscle‐tendon interfaces and, therefore, may contribute to SR.^27^, ^37^

Whereas Eggensperger et al focused on the SH musculature, Dicker et al investigated the morphological changes of the MM and MPM after BSSO.^29^, ^30^, ^32^ A significant decrease in both the volume and maximum cross‐sectional area of the jaw muscles occurred 10‐48 months after BSSO. According to the authors, the two most likely explanations for this finding are as follows: (i) BSSO advancement improves the biomechanics of the masticatory system, and less muscle force is needed for the same masticatory tasks, leading to the adaptational atrophy of the muscles until a new equilibrium is reached; and (ii) the biomechanics have deteriorated, and the impaired function leads to atrophy of the muscles. To investigate the validity of both hypotheses, a second study was performed to evaluate the change in muscle direction and moment arms of bite force, showing that the vertical muscle direction, as well as the moment arms of the MM and MPM, changed post‐operatively, but only after bimaxillary surgery.^32^ However, the clinical relevance of this finding remains unclear in this paper.^32^ Therefore, the same data were used to generate a biomechanical model, allowing assessment of the muscle forces on the condyle and the corresponding joint reaction force. This study showed only minor increases in joint reaction force after surgery, making it unlikely that increased joint loading resulting from changes in muscular direction is a causal factor in the occurrence of SR due to progressive condylar resorption.^29^

### 4.3 Functional changes of the perimandibular muscles

Alternations in the functional properties of a neuromusculoskeletal system are often evaluated using EMG and/or kinesiometry. EMG enables the quantification of muscle activation. The magnitudes of the EMG signals change as the neural signalling calls for increased or decreased muscular effort. Although muscle contraction is initiated by neural muscular activation, the resulting movement and generated forces are further determined by contraction dynamics (depending on the kinetics of the joint) and musculoskeletal geometry (determining the moment arms of the different muscles in the system).^38^

Wessberg and Epker found that the modified sagittal split technique, as described by Epker,^34^ does not significantly influence the masticatory musculature, as the neuromuscular equilibrium is not altered.^25^ However, in 1995, Eckardt et al^24^ concluded that normalization of the EMG to an eugnathic pattern occurs in class II subjects. The most plausible explanation for these contradictory findings is the difference in EMG registration. Wessberg and Epker^25^ used bipolar EMG registration, whereas Eckardt et al^24^ measured EMG activity using 16 unipolar electrodes, enabling registration and comparison of the overall excitation pattern instead of comparing absolute values. Therefore, this method is suitable for overcoming interindividual variation in absolute measurements of EMG activity, which has been shown to be significant with the protocol used by Wessberg and Epker.^39^

Di Palma et al concluded that the improvement in the symmetrical distribution of neuromuscular activity was due to improved stability of the occlusion rather than biomechanical advantages, as the effect was independent of the type of surgical jaw displacement.^26^ However, it should be emphasized that these conclusions were drawn from statistically insignificant results and in contradiction with the differences in excitation patterns and neuromuscular adaptation in Angle class II subjects compared to Angle class III subjects as reported by Eckardt et al.^24^

Based on the limited available evidence in this review, it can be concluded that functional adaptation in terms of EMG activity occurs in the masticatory muscles after BSSO advancement. However, the net result of these changes on the functional properties of the neuromusculoskeletal system cannot be determined as other variables, as described in the first paragraph of this section, might also be affected by orthognathic surgery. Furthermore, it should be emphasized that the relevance of altered EMG activity in the occurrence of SR remains unclear.

### 4.4 Limitations of available evidence

The first limitation of the currently available evidence presented in this review is the lack of studies that used 3D analyses to assess skeletal jaw movements and relapse. Studies that met the inclusion criteria relied on the 2D measurements of the lateral cephalograms. As 3D analyses are increasingly becoming the clinical standard and with proven higher accuracy, the use of 3D evaluation of mandibular advancement and SR is strongly recommended for future studies.^2^, ^3^, ^40^

Another limitation is the lack of specific subject selection in the majority of studies. Instead of including a general group of class II subjects, it is recommended to include a homogenous subject population that is at risk for SR in order to evaluate potential preventive strategies for SR in future studies.

Lastly, the high heterogeneity of treatment protocols (Table 2) and the reported outcomes regarding morphological and functional changes of the perimandibular muscles (Table 5) did not permit a quantitative meta‐analysis.

## 5 CONCLUSION

In conclusion, the findings of this systematic review have demonstrated that the perimandibular muscles play a role in the occurrence of SR after the advancement of BSSO and may serve as a potential target for the prevention, or at least reduction, of SR in specific subjects. Nevertheless, future research is necessary to (i) develop a better understanding of the role of each muscle group, (ii) develop new therapeutic strategies and (iii) define criteria that allow the selection of subjects that will benefit from these new therapies.

## CONFLICTS OF INTEREST

The authors have nothing to declare with regard to conflicts of interest.

## AUTHORS’ CONTRIBUTION

Maxim Van den Bempt: conceptualization; methodology; investigation; visualization; writing – original draft preparation, review and editing. Shankeeth Vinayahalingam: investigation; writing – review and editing. Michael D. Han: writing – review and editing. Stefaan J. Bergé: conceptualization; writing – review and editing; supervision. Tong Xi: conceptualization; methodology; validation; writing – review and editing; supervision.

## ETHICAL APPROVAL

Not required.

## PATIENT CONSENT

Not required.

## Data Availability

The data that support the findings of this study are available from the corresponding author upon reasonable request.
